# Chemotactic Interactions of *Scenedesmus* sp. and *Azospirillum brasilense* Investigated by Microfluidic Methods

**DOI:** 10.1007/s00248-024-02366-3

**Published:** 2024-03-18

**Authors:** Erika Greipel, Krisztina Nagy, Eszter Csákvári, László Dér, Peter Galajda, József Kutasi

**Affiliations:** 1 Albitech Biotechnological Ltd, Berlini Út 47-49, 1045 Budapest, Hungary; 2 Department of Plant Anatomy, ELTE Eötvös Loránd University, Pázmány Péter Stny 1/C, H–1117 Budapest, Hungary; 3 Institute of Biophysics, HUN-REN Biological Research Centre, Szeged, Temesvári Krt. 62, 6726 Szeged, Hungary; 4 Division for Biotechnology, Bay Zoltán Nonprofit Ltd. for Applied Research, Derkovits Fasor 2, 6726 Szeged, Hungary

**Keywords:** Chemotaxis, Chemical gradients, Microfluidics, Microbial communication, Microbial interaction

## Abstract

The use of algae for industrial, biotechnological, and agricultural purposes is spreading globally. *Scenedesmus* species can play an essential role in the food industry and agriculture due to their favorable nutrient content and plant-stimulating properties. Previous research and the development of *Scenedesmus*-based foliar fertilizers raised several questions about the effectiveness of large-scale algal cultivation and the potential effects of algae on associative rhizobacteria. In the microbiological practice applied in agriculture, bacteria from the genus *Azospirillum* are one of the most studied plant growth-promoting, associative, nitrogen-fixing bacteria. Co-cultivation with *Azospirillum* species may be a new way of optimizing *Scenedesmus* culturing, but the functioning of the co-culture system still needs to be fully understood. It is known that *Azospirillum brasilense* can produce indole-3-acetic acid, which could stimulate algae growth as a plant hormone. However, the effect of microalgae on *Azospirillum* bacteria is unclear. In this study, we investigated the behavior of *Azospirillum brasilense* bacteria in the vicinity of *Scenedesmus* sp*.* or its supernatant using a microfluidic device consisting of physically separated but chemically coupled microchambers. Following the spatial distribution of bacteria within the device, we detected a positive chemotactic response toward the microalgae culture. To identify the metabolites responsible for this behavior, we tested the chemoeffector potential of citric acid and oxaloacetic acid, which, according to our HPLC analysis, were present in the algae supernatant in 0.074 mg/ml and 0.116 mg/ml concentrations, respectively. We found that oxaloacetic acid acts as a chemoattractant for *Azospirillum brasilense*.

**Supplementary Information:**

The online version contains supplementary material available at 10.1007/s00248-024-02366-3.

## Introduction

Nowadays, the essential role of microalgae in global carbon and nitrogen cycling, both in marine and freshwater environments, is recognized. Different types and forms of algal biomass have been used for various agricultural purposes, such as food sources, fodder, soil fertilizer, or biostimulants [1–4]. Algae serve as renewable resources for biofuels [5–7] and are promising candidates for diverse pharmaceutical and cosmetic applications e.g., [8–10]. They are a great source of pigments, vitamins, fatty acids, and proteins of interest [11, 12]. Besides the various industrial applications, green algae are also widely studied for their potential usage in water management [13]. The members of the *Scenedesmus* genus have also been studied for such applications in recent years [14].

Besides algae, bacteria are also essential members of the global carbon cycle since they have an important role in decomposing organic matter. Furthermore, certain species of bacteria are also capable of promoting plant growth by various mechanisms (mainly by improving nutrient absorption in the soil) [15]. Microalgae and bacteria influence ecosystems together, and their consortia are shaped by different kinds of interactions (from mutualistic to parasitic). This complex relationship also affects the (co)evolution of these microorganisms [16, 17]. Therefore, for successful future biotechnological applications, microalgae-bacteria interactions have to be considered. A more thorough understanding of the nature and possible controllability of these interactions can be helpful, e.g., in controlling algal blooms or increasing the efficiency of microalgae biomass production and related valuable compounds [16–18].

Studies on microalgae-bacteria interactions revealed that some bacteria can enhance the production of algal biomass and associated compounds by, e.g., secreting growth-promoting factors [19]. One of the most studied plant growth-promoting bacterium (PGPB) is *Azospirillum brasilense*, which can significantly increase algal growth rates through a variety of mechanisms, including the production of indole-3-acetic acid, an auxin hormone [20]. This property of *A. brasilense*, besides the synthesis of nitric oxide, carotenoids, and a range of cell surface components, also contributes to its ability to adapt to the rhizosphere habitat and promote plant growth. It binds N_2_ under microaerobic conditions, denitrifies under anaerobic and microaerophilic conditions, and can assimilate ammonia, nitrate, and nitrite [21].

A well-known method to model and test bacterial-root interaction is to replace higher plants with microalgae [22]. Kim et al. investigated the mutualistic relationship between soil bacteria and different species of green algae [23]. Denaturing gradient gel electrophoresis and 16S rRNA gene clone library experiments with *Chlamydomonas reinhardtii*, *Chlorella vulgaris*, *Scenedesmus* sp*.*, and *Botryococcus braunii* revealed that the dominant phycosphere bacteria hosted by these green algae were *Rhizobium*, *Mesorhizobium*, and *Shinella* within Rhizobiales, *Flavobacterium* within Flavobacteriales, and *Pseudomonas* within Pseudomonadales. When *Rhizobium* sp., isolated from *C. vulgaris*, was co-cultured with green algae, it promoted algal cell count by ∼72%. Likewise, growth rates of algae and *Rhizobium* increased by an average of ∼11% and ∼110%, respectively, confirming mutualistic interactions. Other studies also suggest that microalgae cultures can influence the growth rate of PGPB. Fingerhut et al. studied the effect of *Scenedesmus obliquus* hot water extract on the growth of *Rhizobium japonicum* soil bacteria [24]. Their results showed that increasing the concentration of the algae extract up to 1% in the culture media promoted the growth of the bacterium while adding yeast extract (at concentrations above 0.1%) inhibited it.

The chemotactic behavior of bacteria plays a crucial role in rhizobial and algae-bacteria interactions [25]. Motility and chemotaxis could provide a competitive advantage to bacteria colonizing different niches. In many bacteria, motility is powered by flagella. *A. brasilense* has one polar flagellum for swimming and chemotaxis and several peritrichous flagella for swarming on solid or semi-solid surfaces [26]. As a polar-flagellated bacterium, its main swimming pattern is run and reverse. However, it also performs run-pauses and run-reverse-flick patterns [27]. Several structural components, such as chemotaxis proteins have already been identified that participate in the regulation of the swimming pattern. However, our knowledge of the molecular mechanisms behind the lifestyle of this soil organism [26–29] is still incomplete. *A. brasilense* performs aerotaxis, redox taxis, taxis to alternative electron acceptors, and chemotaxis to oxidizable substrates. The signals for these responses are triggered by changes in the electron transport system [30]. Moreover, Reinhold et al. [31] showed differences in the chemotactic behavior of *Azospirillum* strains isolated from different host plants. The reason behind this could be that plants might differ in their primary photosynthetic pathways: In C_4_ plants, organic acids containing four C atoms, such as malate, are the initial stable product, whereas in C_3_ plants, glycerate-3-phosphate is produced. They found that the wheat (C_3_) isolate was strongly attracted to D-fructose, L-aspartate, citrate, and oxalate, while the other strains, isolated from C_4_ plants, showed maximal attraction to L-malate. They also found that chemotaxis depends on the attractant concentration and stereoconfiguration.

de-Bashan and Bashan proposed an experimental model based on the joint immobilization of microalgae and PGPB in small alginate beads to study plant-bacteria interactions [21, 22]. They studied the interaction of the green microalga *Chlorella vulgaris* and the PGPB *A. brasilense* in this system. Using scanning electron microscopy, they could follow the formation of microcolonies of each microorganism in the polymer beads over several days. Although the immobilization of the cells was initially random within the confinement of the beads, over time, the bacterial and microalgal microcolonies merged and formed large, mixed clusters within the cavities. Since *A. brasilense* is a highly motile bacterial strain with a chemotactic system, the authors suggested that motile bacterial populations moved toward the immobile *C. vulgaris* colonies through the cavities in the alginate beads [32]. The nutrients and the molecules excreted by bacteria (e.g., indole-3-acetic acid) or algae can freely diffuse in the porous gel. These studies suggest that microalgae respond to the interaction with *Azospirillum* similarly to higher plants, with enhanced growth and increased glutamine synthetase and glutamine dehydrogenase enzyme activity [21]. A positive effect of joint immobilization on the *A. brasilense* cell number was also measured [22]. Combining this method with fluorescent in situ hybridization revealed cluster-type interactions in which bacteria and microalgae were bound to each other and the inert matrix via fibrils and sheath material, creating a solid (biofilm-like) structure [22].

Microfluidic technologies provide the tools to mimic the spatiotemporal complexity of natural microhabitats [33]. For example, chemical heterogeneities can be modeled by establishing and controlling chemical gradients on a microscopic scale [34]. A variety of microfluidic devices have been created and used to study bacterial chemotaxis over the past decades [35]. Among these, flow-free gradient generators (relying solely on diffusion) offer the possibility to study chemotactic response sensitively over an extended period of time in a steady liquid medium.

In this work, we used a flow-free, diffusion-based microfluidic setup [36] to study the chemotactic interactions between *Scenedesmus* sp. and *A. brasilense*. Microalgae and bacterial cultures were loaded in microchambers separated by a porous membrane. This membrane served as a physical barrier for the populations but allowing chemical coupling between them. The free diffusive transport of secreted products through the membrane resulted in chemical heterogeneities within the bacterial microhabitat. We hypothesized that even a young 5-day-old algal culture may produce some metabolites that are attractive to the bacteria. The growth and spatial distribution of the *A. brasilense* population were monitored over time by fluorescence time-lapse microscopy. Besides studying the effect of an adjacent microalgae population, the chemoeffector potential of compounds detected in the 5-day-old *Scenedesmus* sp. culture was also tested using the same microfluidic setup.

## Materials and Methods

### Algal Strain and Cultivation

The *Scenedesmus* sp. BEA D01_12 strain [37] was isolated from a shallow freshwater lake (Lake Balaton, Hungary) in May 1995. See the details of the physical and chemical characteristics of the lake water and the composition of phytoplankton (e.g., in [57–59]). The strain was cultured on modified PHM-1 medium which contained 1 g KNO_3_, 0.2 g MgSO_4_, 0.2 g K_2_HPO_4_, 0.1 g sodium acetate, 20 ml saturated CaSO_4_ solution, 10 ml micronutrient solution (61 mg H_3_BO_3_, 38 mg (NH_4_)_6_Mo_7_O_24_ × 4 H_2_O, 6 mg CuSO_4_ × 5 H_2_O, 6 mg Co(NO_3_)_2_ × 6 H_2_O, 2 mg ZnSO_4_ × 7 H_2_O, and 4 mg MnCl_2_ × 4 H_2_O, in 1000 ml distilled water), and 10 ml iron solution (3.8 g Fe(III)NaEDTA × 3 H_2_O in 1000 ml distilled water) in distilled water in a final volume of 1000 ml [38]. All chemicals were purchased from Molar Chemicals (Halásztelek, Hungary), except Fe(III)NaEDTA × 3 H_2_O (Acros Organics, Geel, Belgium). The cultures were grown at 24 °C in 16:8 h light:dark cycles to synchronize the cells. The photon flux density (PFD) was 180 µmol photon m^−2^ s^−1^ (Tungsram FT8/36W/840/GE/LL/SL1/25). The cultivation flasks were shaken at 150 rpm frequency.

### Bacterium Strain and Cultivation

The *A. brasilense* CdS strain used in our experiments produces a green fluorescent protein (GFP) (from the collection of NAIK-ABI made by É. Kárpáti). It was cultured in Erlenmeyer flasks at 28 °C in a medium containing 5 g malic acid, 3 g NaOH, 1 g (NH_4_)_2_SO_4_, 0.007 g FeSO_4_ × 7H_2_O, 0.01 g Na-EDTA, 100 ml 10 × Okon stock solution (2 g MgSO_4_ × 7H_2_O, 1 g NaCl, 0.2 g CaCl_2_ × 2H_2_O, 0.01 g MgSO_4_ × 7 H_2_O, 0.02 g MnSO_4_ × 4H_2_O in 1000 ml distilled water), 100 ml 10 × K-PO_4_ stock solution (40 g KH_2_PO_4_ and 60 g K_2_HPO_4_ in 1000 ml distilled water), 1 ml biotin stock solution (1 mg/ml distilled water), and 800 ml distilled water [39] supplemented with 20 µg/ml of chloramphenicol (Sigma-Aldrich, St. Louis, MO, USA). All chemicals except chloramphenicol were purchased from Molar Chemicals (Halásztelek, Hungary). Before each experiment, the bacterial culture was centrifuged twice (5 min, 3000 rpm), and cells were suspended in PHM-1 algae nutrient solution, a widely used culture media for algae, and here, this constituted the experimental medium. In control experiments carried out with homogeneous concentrations of organic acids, bacteria were centrifuged and suspended in PHM-1 containing citric acid or oxaloacetate.

### Analytical Method

The HPLC system used for the analysis of low molecular weight carboxylic acids from centrifuged supernatant of 5-day-old *Scenedesmus* sp. culture was a Waters 2690 separation module consisting of a low-pressure quaternary mixing valve, a high-pressure pump, a 100 µl capacity auto-injector, and a Jetstream column thermostat. The system was equipped with a Waters 2690 diode array detector with 208-nm monitoring wavelength. The system hardware, HPLC data acquisition, and data processing were controlled by the Millennium 4.0 software. The applied stationary phase was a 250*4.6 mm Alltima-C18 column filled with 5-µm-diameter spherical particles. The isocratic mobile phase consisted of 9 volumes of 50 mM K_3_PO_4_ with 10 w% H_3_PO_4_ and 1 volume of MeOH. The flow rate was maintained at 1.0 ml/min for the overall 15 min chromatographic run duration. The chromatographic column was thermostated to 30 °C temperature; 20-µl sample injection was adapted to achieve the optimal sensitivity. Calibration was made by 1.25 mg/ml solutions of malic acid, citric acid, oxaloacetic acid, succinic acid, and fumaric acid (all from Molar Chemicals, Halásztelek, Hungary) in distilled water.

### Microfluidic Setup

**Fig. 1:**
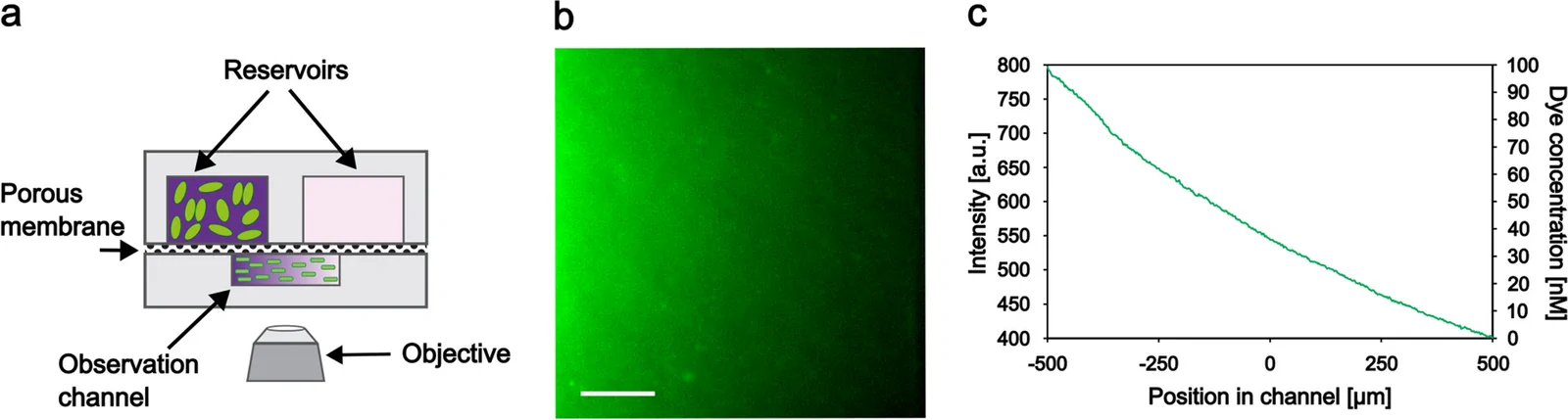
The microfluidic setup to study the chemotactic behavior of *A. brasilense*. (**a**) Schematic drawing of the microfluidic gradient generator device (cross-sectional view, not scaled). (**b**) Fluorescence microscopy image of pyranine gradient in the observation channel. 100 nM pyranine was loaded into the left reservoir, and pure phosphate buffer was loaded into the right one. The scale bar is 200 µm. (**c**) Average intensity and concentration profile taken across the middle area of the observation channel in the presence of fluorescent dye gradient

The microfluidic gradient generator device [36] was made of poly-dimethylsiloxane (PDMS). It consists of two large chambers (reservoirs) and an observation channel that are separated by a porous membrane (Whatman, Anodisc, diameter 47 mm, pore size 0.1 µm). Due to the membrane’s pore size, the molecules can freely diffuse between the chambers and the channel, but the cells do not pass through it. By adding a chemoeffector solution to one of the reservoirs, a linear chemical concentration gradient forms in the observation channel. The schematic drawing of the experimental setup is presented in Fig. 1a. Gradient formation was demonstrated by using pyranine, a water-soluble fluorescent dye solved in potassium-phosphate buffer (pH 7.0) in a 100-nM maximum concentration (Fig. 1b,c). Intensity data were converted into concentration values based on a control experiment, where pyranine was loaded in the same concentration into both reservoirs.

Bacteria were loaded into the observation channel, and the chemotactic response of the population was followed by fluorescence time-lapse microscopy. Throughout the experiments, we always added the potential chemoeffectors or conditioned media into the left side reservoir, while the right side contained pure PHM-1. Citric acid monohydrate and the sodium salt of oxaloacetate were solved in PHM-1 to reach 0.4 mM and 0.8 mM concentrations of citric acid and oxaloacetate, respectively (Molar Chemicals, Halásztelek, Hungary). In the case of co-culture studies, the reservoir on the left side was filled with *Scenedesmus* sp. culture, and the response of *A. brasilense* cells was monitored in the adjacent observation channel. To prevent cell adhesion, 10 mg/ml bovine serum albumin was applied in each solution.

### Microscopy and Image Analysis

The experiments were performed using a Nikon Eclipse Ti-E inverted microscope, 10 × Nikon Plan Fluor lens, GFP fluorescence filter (49,002 filter set, Chroma Inc.), and LUMEN 200Pro excitation lamp (Prior Scientific Ltd.). The imaging equipment was an Andor NEO sCMOS camera (Andor Technology plc.). The microscope and the camera were controlled using NIS Elements Advanced Research (Nikon Inc.) software. Photos were taken every 5 min.

An open-source software package, ImageJ, was used for image analysis and data processing [40]. A background correction, based on the “rolling ball” algorithm, was applied to each fluorescence image. To quantify the spatial distribution of bacteria across the width of the observation channel, we used the parameter “asymmetry index” (*A*). To calculate *A*, first, we divided the channel into two equal parts (left and right) and measured the average fluorescence intensity in each part (*I*_left_ and *I*_right_). Then, we calculated* A* by the following formula:

1 $$A=\frac{{I}_{{\mathrm{left}}}-{I}_{{\mathrm{right}}}}{{I}_{{\mathrm{left}}}+{I}_{{\mathrm{right}}}}$$

The value of *A* varies between − 1 and 1. *A* is positive when most cells are on the left side of the observation channel and negative when most cells are on the right side of the channel. Zero corresponds to an evenly distributed cell population. Its temporal change illustrates the dynamic rearrangement of the bacterial population in the channel. The calculated average intensity values were also plotted as the function of time to quantify and present population growth in the observation channel.

The average fluorescence intensity profiles across the width of the observation channel show the distribution of the cells at characteristic time points (0 min and 9 h). Kymographs were also created from the fluorescence image series by vertically compressing each frame and stacking them along the *y*-axis.

Inkscape, a free, open-source vector graphics editor, was used to prepare the figures.

## Results

We examined the chemotactic behavior of *A. brasilense* soil bacteria toward a neighboring *Scenedesmus* sp. microalgae population. To determine whether the compounds secreted by the algae have any chemoeffector potential, we used a microfluidic device, which allowed the formation of stable linear chemical concentration gradients. The schematic drawing of the gradient generator device is shown in Fig. 1a. The linear chemical concentration gradient across the width of the channel can be represented by adding a fluorescent dye solution in one of the reservoirs (the left one in Fig. 1b,c).

### *A. brasilense* and *Scenedesmus* sp. in Chemically Coupled Microchambers

**Fig. 2:**
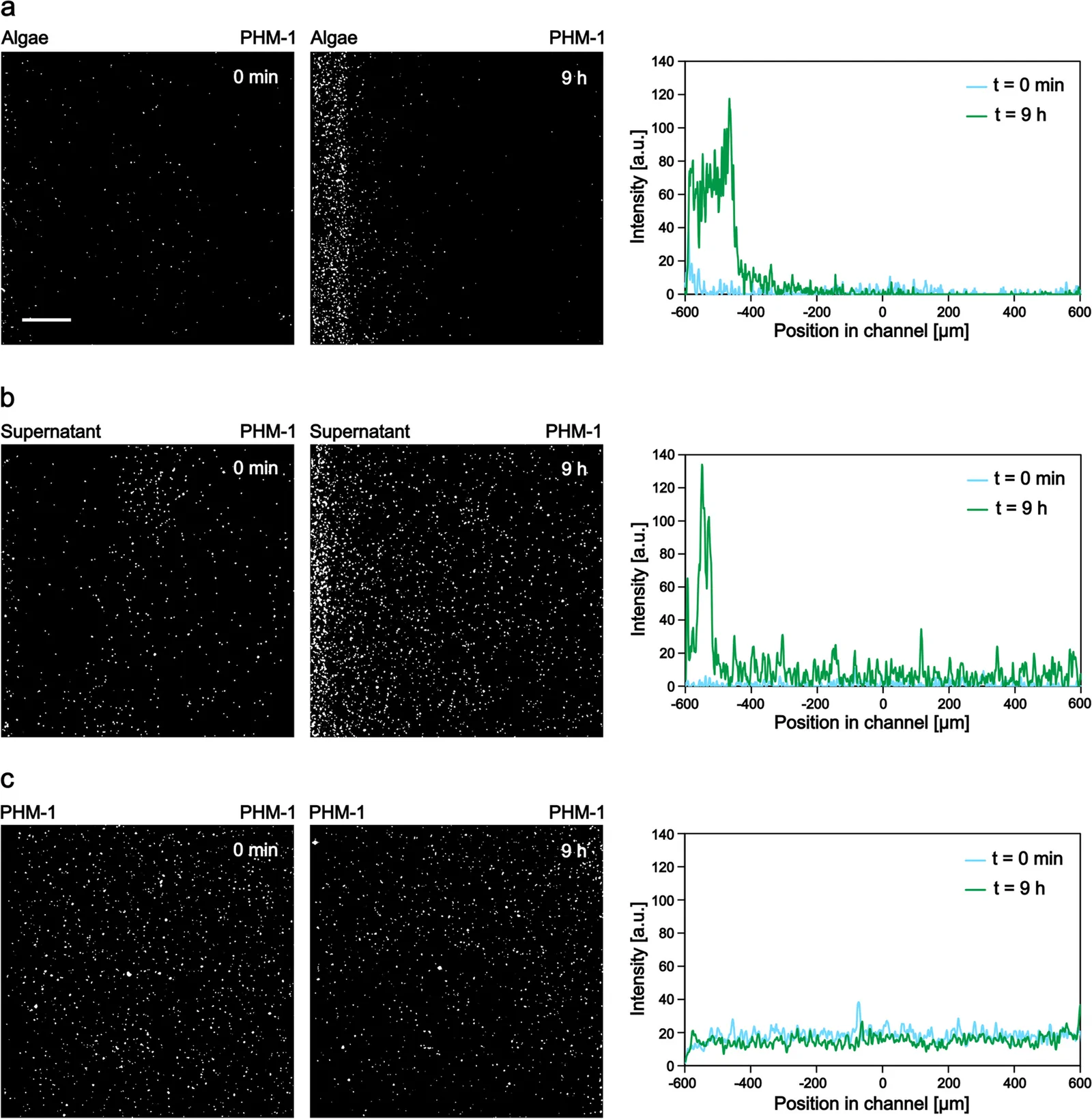
Chemotactic response of *A. brasilense* toward *Scenedesmus* sp. Characteristic images taken at the beginning and at the end (9 h) of experiments, together with the corresponding average intensity profiles, show the distribution of cells in the observation channel. The scale bar is 200 µm. (**a**) Co-culture experiment, where algae culture was loaded into the left reservoir and PHM-1 medium into the right one. (**b**) The cell-free supernatant of the centrifuged algae culture was loaded into the left reservoir and PHM-1 into the right one. (**c**) Control experiment where PHM-1 was applied in both reservoirs

As a first step, we investigated the behavior of bacteria against an adjacent microalgae culture (Fig. 2a). For this purpose, we loaded a 5-day-old *Scenedesmus* sp. culture into the left-hand side reservoir of the device and a fluorescently labeled *A. brasilense* CdS culture into the observation channel (the same way as it is presented in Fig. 1a). The communication between the two microbial populations was facilitated by the porous membrane between the microchambers. The spatial distribution and proliferation of bacterial populations in the observation channel were monitored over time by fluorescence time-lapse microscopy.

In these experiments, we consistently observed that bacteria gathered on the algae culture side (left) of the observation channel, as demonstrated by fluorescence images and the corresponding intensity profiles in Fig. 2a. In some experiments, we observed groups of bacteria swimming toward the algae culture, which suggests the importance of directed active movement (chemotaxis) in the observed accumulation of bacteria. These chemotactic waves took place within the first 3–4 h of the experiments. A typical example of such a response is presented in Supplementary Fig. 1b, kymograph #1. The two-sided distribution of cells at the beginning of this experiment resulted from the filling process. However, we can clearly see that the subpopulation of bacteria on the right moves toward the algal culture (left side of the channel) within a few hours. In another example (presented in Supplementary Fig. 1b, kymograph #2), we started with a homogeneous distribution of bacteria. In this case, the formation of a chemotactic wave was lacking, but nevertheless, cells accumulated over time next to the algae culture in the channel.

Besides chemotaxis, differential growth might also lead to the accumulation of cells on the left side of the observation channel. A location-dependent growth rate due to the spatial gradient of nutrients produced by the algae might lead to an uneven distribution of bacterial cells within the channel. Such differential growth can be seen, e.g., in the increment of the average intensity values in Fig. 2a (right panel) or in the kymographs (Supplementary Fig. 1b). Both chemotaxis and differential growth might shape the population’s distribution across the width of the microchannel.

Experiments carried out with algae-conditioned media (cell-free supernatant of centrifuged algae culture) resulted in similar bacterial behavior (Fig. 2b, Supplementary Fig. 1c). We observed both the chemotactic waves (kymograph #1) and the differential growth of the bacteria during these experiments (Supplementary Fig. 1c, kymographs #1 and #2). Control experiments without any gradients (PHM-1 medium in both reservoirs) demonstrated a homogeneous distribution of the bacterial population over the experiment (Fig. 2c, Supplementary Fig. 1a). Furthermore, no intense growth was observed during the control experiments. Fluorescence microscopy snapshots of bacteria from characteristic experiments are presented in Fig. 2, together with the average intensity profiles taken across the width of the channel.

**Fig. 3:**
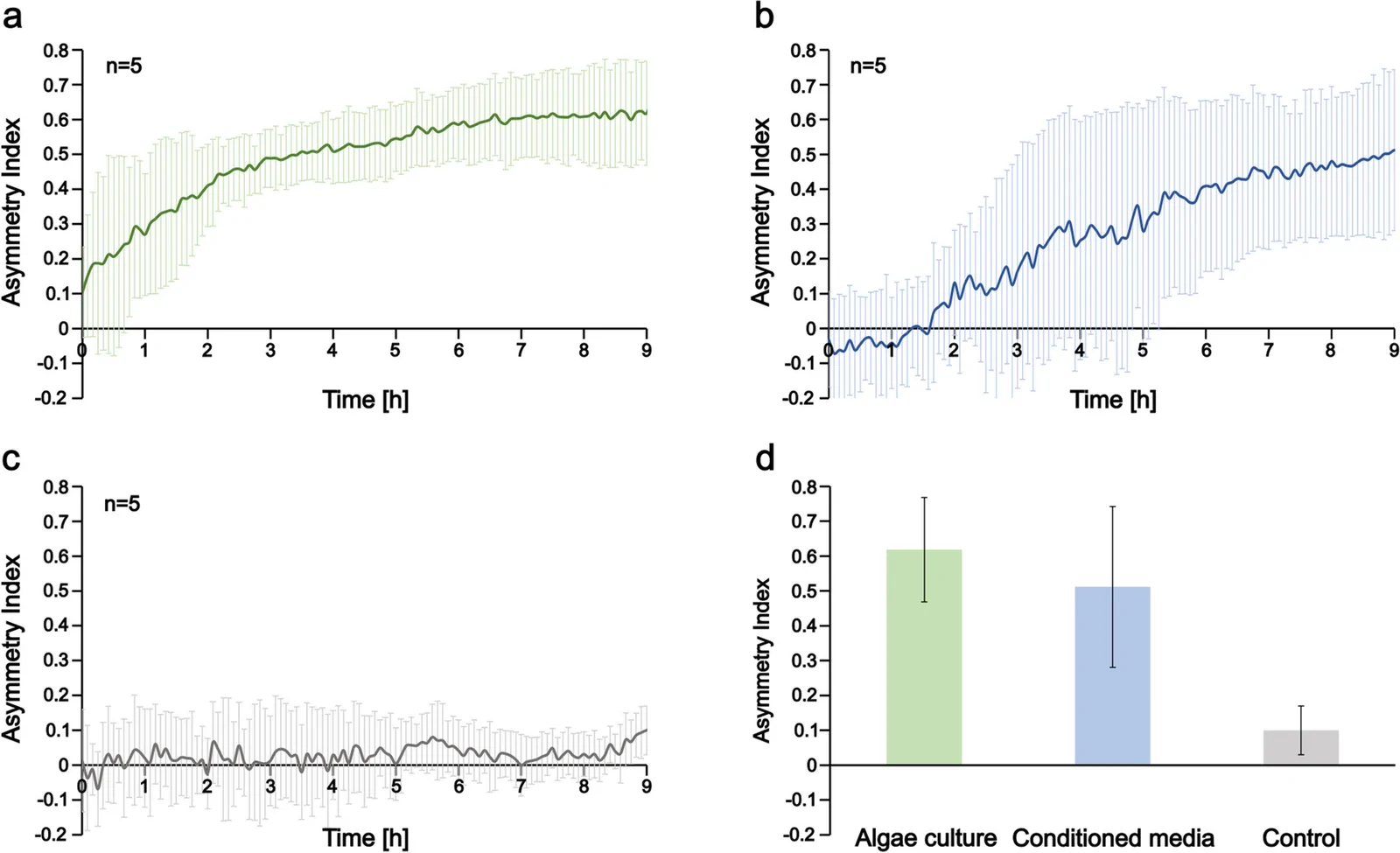
Asymmetry index (*A*) shows the temporal change of the spatial distribution of *A. brasilense* CdS cells across the observation channel in the presence of an adjacent *Scenedesmus* sp. population and in control experiments. (**a**) Gradient was formed by inoculating algae culture into the left reservoir and PHM-1 into the right one. (**b**) Conditioned media (supernatant of algae culture) was loaded into the left and PHM-1 medium into the right side of the device. (**c**) Control experiments where cells were not exposed to any gradient, but PHM-1 algae medium was loaded into both reservoirs. (**d**) Bar graphs representing the calculated *A* values at the end of the experiments (9 h of incubation in the microfluidic device)

To quantitatively describe the spatial distribution of bacteria across the width of the observation channel, we calculated the asymmetry index (*A*) according to Eq. (1). Changes in *A* give us information on the spatial population dynamics. In the case of algae culture or its conditioned media in the left reservoir, *A* increased during the first 4–5 h as cells accumulated on the left side of the channel (Fig. 3a,b). Then, it saturated at a value of 0.5–0.6. In control experiments, where bacteria exhibited a nearly homogeneous spatial distribution, *A* was found to be near zero (Fig. 3c) throughout the experiments. After 9 h of incubation in the device, we calculated 0.62 ± 0.15, 0.5 ± 0.23, and 0.1 ± 0.07 values for *A* in case of loading algae culture, cell-free conditioned media, and pure PHM-1 into the left reservoir, respectively (Fig. 3d). The right-side reservoir always contained PHM-1 medium.

#### Analytical Measurements of the Conditioned Media

**Fig. 4:**
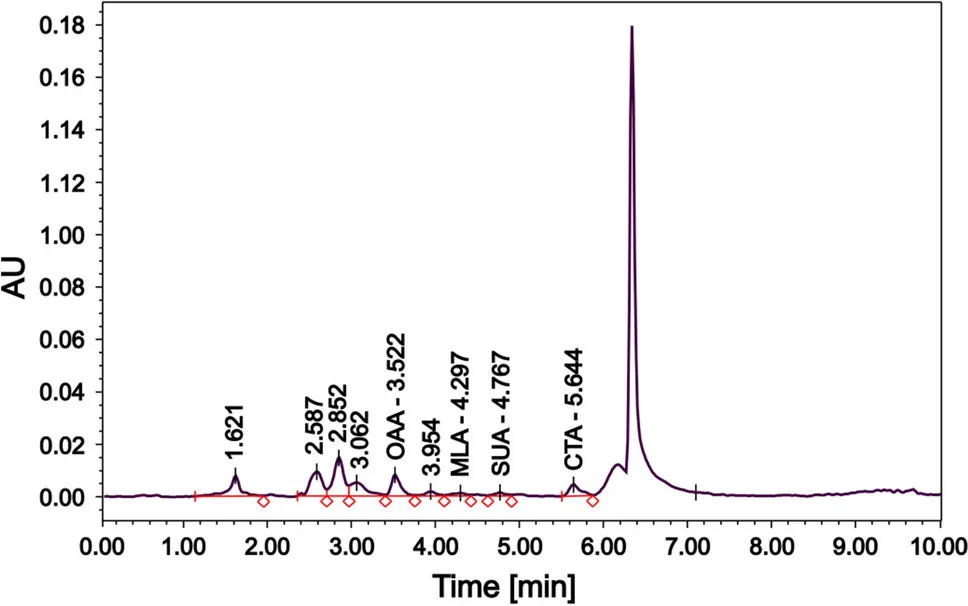
HPLC–PDA chromatogram of the small-size organic acid compounds of the supernatant of the 5-day-old culture of *Scenedesmus* sp. in PHM-1 medium. Abbreviations represent oxaloacetic acid (OAA), malic acid (MLA), succinic acid (SUA), and citric acid (CTA)

To identify potential chemoeffectors produced by the algal cells, we analyzed the low molecular weight organic acid content of the *Scenedesmus* sp. culture by HPLC chromatography. The chromatogram and the quantitative results are shown in Fig. 4 and Table 1, respectively.

**Table 1:** Quantitative results of HPLC–PDA measurement of low molecular weight organic acids in the supernatant of the 5-day-old *Scenedesmus* sp. culture

Sample	Peak name	Retention time (min)	Area	% Area	Concentration (mg/ml)
Calibration solution	Oxaloacetic acid	3.55	1,192,379	13.45	1.250
	Malic acid	4.23	3,014,323	34.00	1.250
	Succinic acid	5.12	2,305,300	26.01	1.250
	Citric acid	5.83	771,433	8.70	1.250
	Fumaric acid	7.28	1,581,112	17.84	1.250
Algae supernatant	Oxaloacetic acid	3.52	62,748	12.25	0.116
	Malic acid	4.30	14,492	2.83	Cannot be determined
	Succinic acid	4.78	13,278	2.59	Cannot be determined
	Citric acid	5.64	42,618	8.32	0.074

We selected five different organic acids (oxaloacetic acid, malic acid, succinic acid, citric acid, and fumaric acid) and tested whether they were present in the supernatant of the 5-day-old algae culture. These compounds are part of the tricarboxylic acid (TCA) cycle. Therefore, we hypothesized that some of these acids could be found even in the young algae culture. Furthermore, according to the literature, such low molecular weight organic acids might behave as chemoeffectors for *A. brasilense* [41]. Based on the analytical measurements, oxaloacetic and citric acids were found to be potential candidates for the induction of the observed chemotactic response of bacteria, as they were present in the supernatant in 0.116 mg/ml (0.9 mM) and 0.074 mg/ml (0.4 mM) concentrations, respectively (Table 1). The other three acids were not detectable in the supernatant.

#### Chemoeffector Potential of Low Molecular Weight Carboxylic Acids

**Fig. 5:**
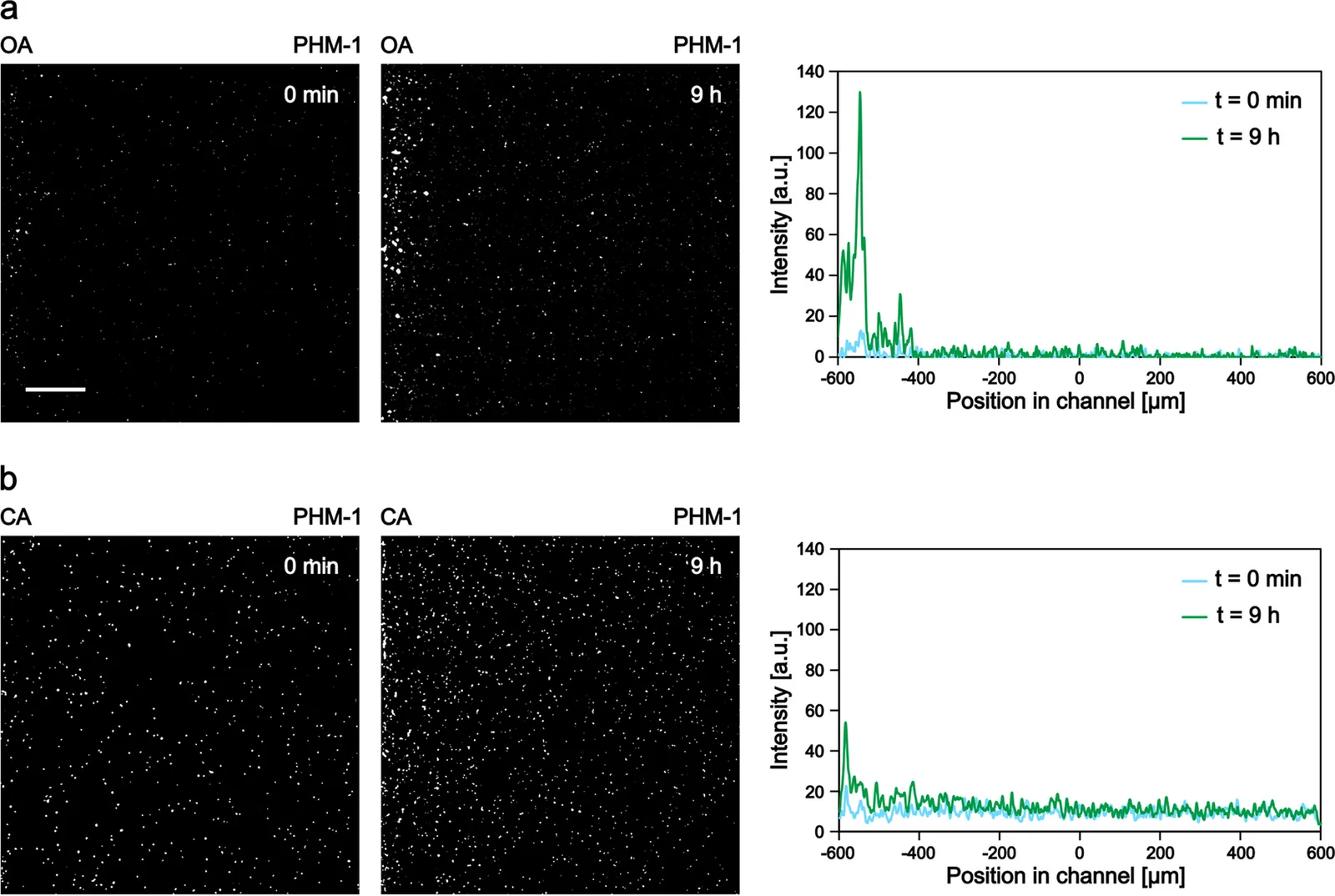
Fluorescence microscopy images and the corresponding average intensity profiles in the presence of low molecular weight organic acid gradients. (**a**) Oxaloacetate (OA) is loaded into the left reservoir and PHM-1 medium into the right one. (**b**) Citric acid (CA) is loaded into the left reservoir and PHM-1 medium into the right one. The scale bar is 200 µm

**Fig. 6:**
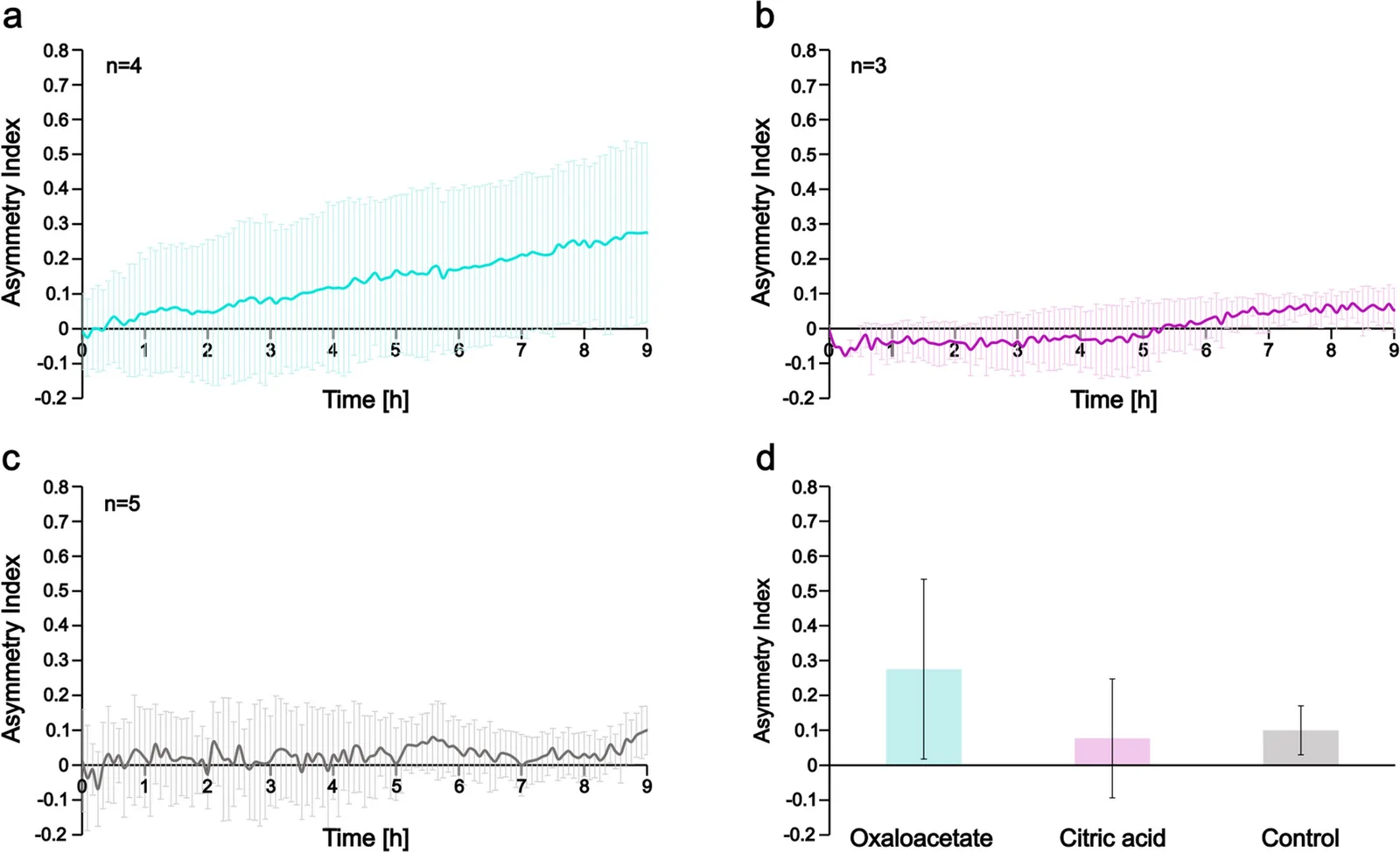
Asymmetry index (*A*) shows the temporal change of the spatial distribution of *A. brasilense* cells across the channel in the presence of organic acid gradients. (**a**) Oxaloacetate was loaded into the left reservoir and PHM-1 into the right one. (**b**) Citric acid was added into the left and PHM1 into the right reservoir. (**c**) Control experiments where cells were not exposed to any gradient (PHM-1 in both reservoirs). (**d**) Asymmetry index (*A*) values were calculated at the end of the experiments (9 h of incubation)

We tested the chemoeffector potential of the detected low molecular weight organic acids on the *A. brasilense* CdS strain by creating linear concentration gradients of these chemicals. For this purpose, citric acid monohydrate and the sodium salt of oxaloacetate were solved in PHM-1 medium to reach the desired concentration of the organic acids: 0.4 mM in the case of citric acid (CA), and 0.8 mM in the case of oxaloacetate (OA). These solutions were loaded into the left reservoir and the behavior of bacteria in the observation channel was followed by fluorescence time-lapse microscopy. Characteristic images and the corresponding average intensity profiles are shown in Fig. 5. In the case of oxaloacetate, we detected small-scale accumulation and growth of bacteria next to the left reservoir. This is also represented in the increased peak of the average intensity profile (Fig. 5a) and can be followed in a kymograph of a characteristic experiment shown in Supplementary Fig. 2a. The average asymmetry index calculated for these experiments also showed a slight increase in time. At the end of the 9-h incubation, its value was 0.28 ± 0.25 (Fig. 6a,d).

In the case of citric acid, the detected effect was not remarkable (Fig. 5b and Supplementary Fig. 2b), and the calculated Asymmetry index was very similar to the one observed in the control experiments (Fig. 6b,d). It is around zero during the experiments, corresponding to a quasi-homogeneous distribution. Its value is 0.08 ± 0.17 at the end of the 9-h-long experiments. Control experiments by applying citric acid (0.4 mM) or oxaloacetate (0.8 mM) in both reservoirs and the observation channel were also performed, and the results are presented in the Supplementary Information. Kymographs and the asymmetry index show a homogeneous distribution of cells in the observation channel during the entire experiment (Supplementary Fig. 2c,d and Supplementary Fig. 3a).

**Fig. 7:**
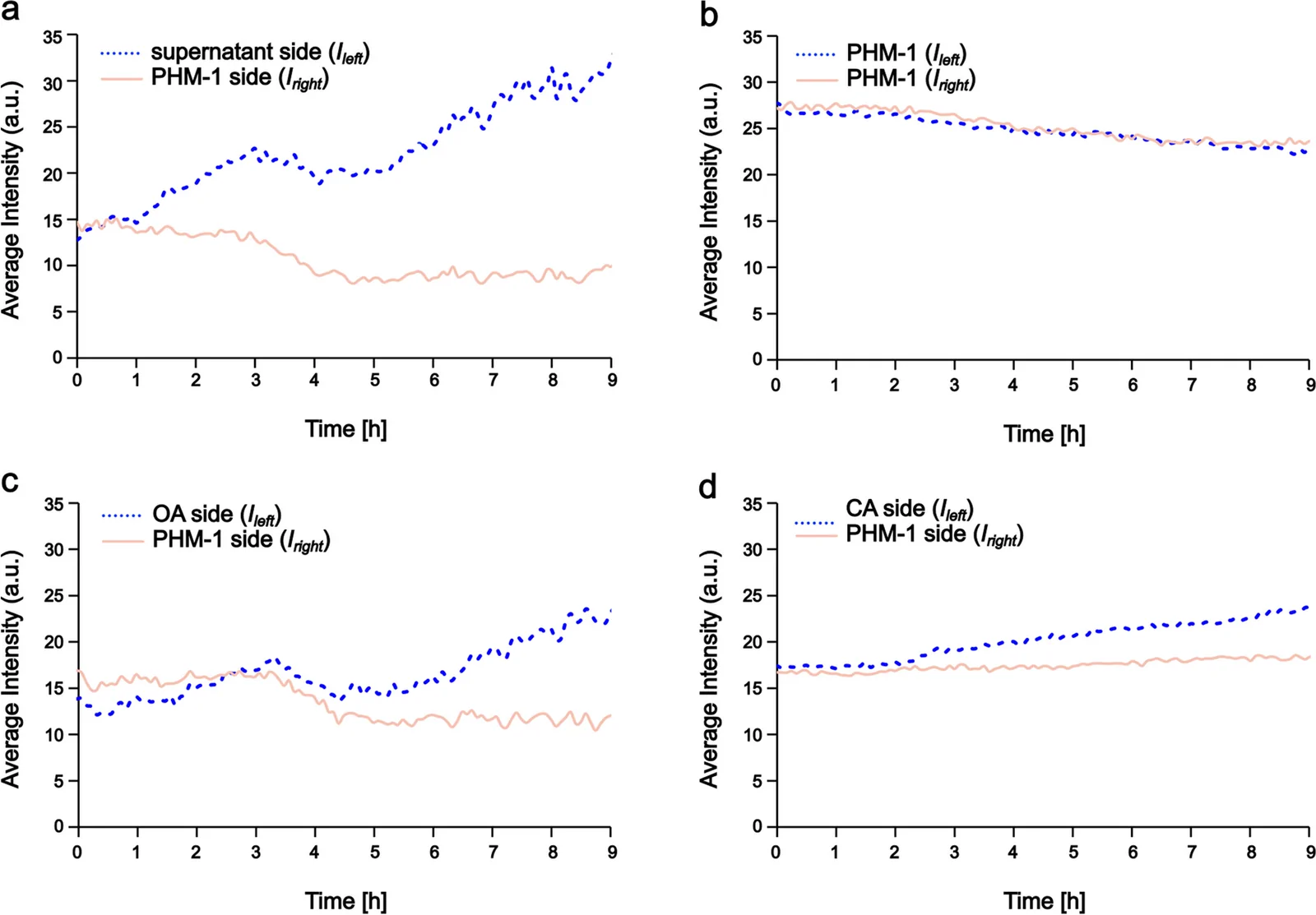
Average fluorescence intensity changes show the growth of the bacteria population in the observation channel (dotted and continuous lines correspond to the average intensity calculated for the left and right half of the microscopy images, respectively). (**a**) The supernatant of the algae culture was loaded for gradient formation in the left reservoir. (**b**) Control experiment performed in PHM-1 media without gradient formation. (**c**) Oxaloacetate (OA) was loaded for gradient formation in the left reservoir. (**d**) Citric acid (CA) was loaded for gradient formation in the left reservoir

The calculated asymmetry index (*A*) presented in Fig. 4 and Fig. 6 gives us information on the distribution of the bacterial population in the observation area, whether cells gather on the chemoeffector side of the channel or not. However, it does not reflect the growth of the population in time. To quantitatively describe how the population size changes over time, we measured the average fluorescence intensity. By dividing the observation channel into two equal parts (left and right, in the same way, it was done when calculating *A*) and measuring the corresponding average intensity values, we can see if there is enhanced growth on the chemoeffector side. Figure 7 shows how these intensities change during the characteristic experiments presented above (in Figs. 2 and 4). No growth was observed on the PHM-1 (right) side of the observation channels, while little growth was seen on the chemoeffector (left) side. The effect was most pronounced in the case of the algal supernatant (Fig. 7a). No growth was detected on either side in the control experiments performed without chemoeffectors (Fig. 7b). In the presence of oxaloacetate, we detected low growth, which was also present in the control experiment when a homogeneous distribution (i.e., no gradient) was applied (Fig. 7c, Supplementary Fig. 3b). In case of citric acid, the growth is negligible both in the presence and absence of gradient (Fig. 7d, Supplementary Fig. 3c).

## Discussion

*A. brasilense* is one of the most studied PGPB. Its motile behavior plays a crucial role in the successful colonization of plant roots [42]. Deepening our knowledge of the chemotactic interactions between green microalgae and PGPB soil bacteria is vital in understanding essential host-bacteria interactions in the rhizosphere. Furthermore, both *A. brasilense* and *Scenedesmus* sp*.* have huge potential in various biotechnological and agricultural applications (e.g., [14, 60]). Understanding the interactions between *Scenedesmus* sp*.* and other microorganisms, especially bacteria, could be essential to increase the biomass production of microalgae and the related compounds (e.g., vitamins and fatty acids).

We used a microfluidic gradient generator device and fluorescence time-lapse microscopy to study the interaction between *Scenedesmus* sp. and *A. brasilense* in detail. This device had been used previously to study the chemotaxis of *Escherichia coli* to different amino acids and signal molecules [36]. Based on earlier results, a spatial chemical gradient was established across the width of the observation channel within minutes, which stayed stable throughout the 9-h timescale of our experiments [36]. Furthermore, it is also suitable for studying the interaction of physically separated but chemically coupled microbial populations [36, 43]. With this experimental setup, we demonstrated the chemotaxis and differential growth of *A. brasilense* in the vicinity of a 5-day-old algal culture and its supernatant. The 5-day-old algal culture we used is considered a young culture in which the presence of lysed cells is not typical. Therefore, cell debris cannot serve as a potential nutrient source for the bacteria in these experiments. By analyzing the small-molecule organic acid content of the algal culture, we detected that oxaloacetic acid and citric acid were present in such young cultures in considerable concentrations. Furthermore, we showed that oxaloacetic acid acts as a chemoattractant for the studied *A. brasilense* CdS strain, which might play a role in the observed accumulation of bacteria close to a neighboring microalgae culture.

Algae (together with lichens) are the primary organic acid producers in the soil ecosystem (in the rhizosphere as well) [44]. The production of low molecular weight organic acids (e.g., citric, oxalic, malic, succinic, fumaric) in these microorganisms is strongly linked to the TCA cycle in the mitochondria. Therefore, it is not surprising to detect these metabolites in well-grown, aging (e.g., 10-day-old) algae cultures in which cellular components derived from dead cells are present. However, in some studies using mammalian cell cultures, TCA cycle intermediates had also been found in the cell-free supernatant at micromolar concentrations [45]. An explanation for this could be that mitochondria can release TCA cycle metabolites to control cell fate and function [46]. In our study, we performed HPLC chromatography to measure the concentration of five different low molecular weight organic acids in the cell-free supernatant of a 5-day-old *Scenedesmus* sp. culture. We found that fumaric acid, malic acid, and succinic acid are absent, but oxaloacetic acid and citric acid are present in the supernatant in 0.9 mM and 0.4 mM concentrations, respectively. To our knowledge, these organic acids have not been previously detected in such young algae cultures.

The above-mentioned low molecular weight organic acids might act as chemoeffectors for different bacteria in the soil ecosystem. It has been shown before that the soil bacteria *Comamonas testosteroni* and *Pseudomonas putida* exhibit positive chemotaxis to oxaloacetate and citrate [47, 48]. The chemoreceptors MCP2201 of *C. testosteroni* and McpS from *P. putida* were shown to bind these molecules [49]. TCA cycle intermediates are abundant in root exudates and plant tissue [50, 51], which might explain the benefit of positive chemotaxis toward these compounds. Furthermore, variations in the chemotactic response of different *Azospirillum* strains may be linked to their host specificity [31], which suggests that the chemotaxis of *Azospirillum* strains can be a consequence of adaptation to the given conditions.

*Azospirillum* has a versatile metabolism. It can use a wide range of carbon sources and has a complete TCA cycle and Entner-Doudoroff pathway [30, 52–54]. There have been attempts to utilize different amino acids, sugars, and organic acids as substrates for cultivating *A. brasilense* strains. Several of the tested organic acids (e.g., malic acid) proved efficient to use in the culture media. In the case of the two chemicals we examined, citric acid failed the test, while oxaloacetic acid was not tested [55, 56]*.* In previous studies, these compounds had been tested for metabolism-dependent chemotactic response in *A. brasilense* and proved to be moderate attractants [30, 41]. The fact that these compounds are part of the TCA cycle suggests that *A. brasilense* might use them as a carbon source. However, we are not aware of any direct evidence that either of these compounds induces significant growth by applying them as the sole carbon source, and they are not typically used in the culture media of *A. brasilense*. In our experiments carried out with the *A. brasilense* CdS strain, we did observe some degree of cell growth, mainly in regions of the observation channel containing oxaloacetic acid. However, the extent of growth measured in the presence of oxaloacetic acid was small, and it was lower than in the presence of the cell-free supernatant of the young algae culture (Fig. 7). This suggests that other compounds, besides the low molecular weight organic acids we found, could also contribute to the observed moderate growth (and chemotaxis). Based on our experiments, citric acid has a negligible effect on the motile behavior and growth of the bacteria population.

Previously, in the literature, the interaction of *A. brasilense* and microalgae (such as *Chlorella* sp.) was studied mainly by co-immobilizing them using alginate beads. In the work of Lebsky et al. [32], in alginate beads, populations of *A. brasilense* swam toward immobile algae colonies, then they merged and formed a larger mixed colony. de-Bashan et al. [22], using the same system, found that *A. brasilense* reached a significantly higher cell number on the 7th day of breeding in case of co-immobilization compared to the control, and the onset of decline also shifted from day 3 to day 10. After the dissolution of alginate beads, the mixed cultures retained the shape of their colonies. Individual cells and homogeneous clusters were observed on the first day of co-culture. This is a complex system where the growth and movement of cells can be followed. However, various nutrients and other chemical gradients affect the bacterial population simultaneously, and the chemical environment might also change during the experiments lasting for several days. Therefore, the details of pure chemotactic responses cannot be studied in this manner. In contrast, microfluidics offers excellent tools to create precise and well-controlled linear chemical concentration gradients. Furthermore, the microfluidic device we use is a flow-free setup that allows bacteria to swim freely in the observation channel and find optimal conditions. The same device can be used for co-culture studies and for testing the chemoeffector potential of selected chemicals [36, 43].

## Conclusion

Co-cultivation with *Azospirillum* species may be a new way of optimizing *Scenedesmus* culturing, but the functioning of the co-culture system still needs to be fully understood. Most studies focus on how bacteria stimulate algal growth (e.g., producing indole-3-acetic acid in the case of *A. brasilense*); however, the effect of algae on the bacteria population is also essential to understand these basic natural interactions between microalgae and bacteria. Our experiments proved that a young, 5-day-old *Scenedesmus* sp. culture already contains chemicals that act as attractants for *A. brasilense*. The strong positive chemotaxis toward the neighboring algae culture happens within hours. We did not use additional nutrients in the media; therefore, it was also surprising that such a young culture (that did not contain lysed cells in a considerable amount) caused differential growth in the bacterial population. Furthermore, based on our work, it can be assumed that organic acids excreted by *Scendesmus* sp*.* play a role in the observed behavior of the bacterial population since oxaloacetic acid (in a similar concentration as we measured in the cell-free supernatant of the algae culture) proved to be a weak chemoattractant for *A. brasilense*. The detected chemotactic response of *A. brasilense* could be relevant not only in microalgae-bacteria interactions but also in the rhizosphere where oxaloacetate (together with other organic acids) is present in root exudates.

## Supplementary Information

Below is the link to the electronic supplementary material.

Supplementary file1 (DOCX 5370 KB)

## Data Availability

No datasets were generated or analysed during the current study.
