# Supplementary material for: Chemotactic Interactions of Scenedesmus sp. and Azospirillum brasilense Investigated by Microfluidic Methods
Source: Microb Ecol. 2024 Mar 18;87:52. doi: 10.1007/s00248-024-02366-3 (PMC10948495; doi:10.1007/s00248-024-02366-3)
Supplement: Supplementary file 1 [file 248_2024_2366_MOESM1_ESM.docx]

Supplementary Information

for

**Chemotactic interactions of *Scenedesmus* sp. and *Azospirillum brasilense* investigated by microfluidic methods**

**Erika Greipel^1,2*^, Krisztina Nagy^3*#^, Eszter Csákvári^3^, László Dér^3^, Peter Galajda^3^, József Kutasi^1^**

^1^Albitech Biotechnological Ltd., H-1045 Budapest, Berlini út 47-49.

^2^Department of Plant Anatomy, ELTE Eötvös Loránd University, H–1117 Budapest, Pázmány Péter stny. 1/c.

^3^HUN-REN Biological Research Centre, Szeged, Institute of Biophysics, H-6726 Szeged, Temesvári krt. 62.

# **^*^**E. Greipel and K. Nagy contributed equally to this work.

^#^Correspondence:

Krisztina Nagy: nagy.krisztina@brc.hu

**This file includes Supplementary Figures 1-3.**

**Supplementary Fig. 1 Kymographs showing the temporal change of the spatial distribution of *A. brasilense* bacteria across the channel.** a) Control experiment without any gradient (PHM-1 medium in both reservoirs). b) Experiments where algae culture was loaded into the left reservoir and PHM-1 into the right one. #1 and #2 show two characteristic population dynamics. c) Experiments where algae-conditioned media was loaded into the left reservoir and PHM-1 into the right one. #1 and #2 show two characteristic population dynamics.

**Supplementary Fig. 2 Kymographs showing the temporal change of the spatial distribution of *A. brasilense* bacteria across the channel in the presence of oxaloacetate (OA) and citric acid (CA).**  a) Response of the bacterial population to the presence of OA gradient (OA is loaded into the left reservoir and PHM-1 into the right one). b) Response of the bacterial population to the presence of CA gradient (CA is loaded into the left reservoir and PHM-1 into the right one). c) The effect of homogeneous OA concentration on the bacterial population. d) The effect of homogeneous CA concentration on the bacterial population

**Supplementary Fig. 3 Temporal change of the spatial distribution and growth of *A. brasilense* CdS population across the observation channel in the presence of homogeneous concentrations of citric acid (CA) and oxaloacetate (OA) within the microfluidic device.** a) Asymmetry index (*A*) in the function of time (dotted line for CA and continuous line for OA). b) Average fluorescence intensity values calculated for the left and right sides of the observation channel in case of homogeneous OA concentration (0.8 mM). c) Average fluorescence intensity values calculated for the left and right sides of the observation channel in case of homogeneous CA concentration (0.4 mM).
